# In vitro probiotic screening and evaluation of space‐induced mutant *Lactobacillus plantarum*

**DOI:** 10.1002/fsn3.1894

**Published:** 2020-09-18

**Authors:** Dan Wang, Tiehua Zhang, Haiqing Ye, Hongwei Hao, Hongxing Zhang, Changhui Zhao

**Affiliations:** ^1^ College of Food Science and Engineering Jilin University Changchun China; ^2^ Fullarton Bioengineering Technology Co., Ltd Beijing China; ^3^ College of Food Science and Engineering Beijing University of Agriculture Beijing China

**Keywords:** lactic acid bacteria, probiotic characteristics, space mutagenesis

## Abstract

Space mutation is an efficient tool in microbial breeding. The aim of the present study was to screen out space‐induced mutants of *Lactobacillus plantarum* with potent probiotic properties. The wild‐type *Lactobacillus plantarum* GS18 was subjected to 31 days and 18.5 hr of space flight, in which 13 isolates were selected for analysis. These mutants were assayed for milk fermentation performance, low pH resistance, bile salt tolerance, hydrophobicity, and antimicrobial activity. The 16S rDNA sequencing was applied to identify the stain and compare it with the wild type. Results showed that the isolate *L. plantarum* SS18–50 had the strongest probiotic properties with no mutation in 16S rRNA sequence compared to the wild type. Specifically, *L. plantarum* SS18–50 had good milk fermentation performance. The viscosity of fermented milk, acid tolerance, and bile salt tolerance were increased by approximately 10%, 8%, and 9%, respectively (*p* < .05). The antibacterial activity (*Escherichia Coli, Salmonella Typhimurium, and Listeria Monocytogenes*) was also increased significantly compared to the wild type (*p* < .05). This study indicates that *L. plantarum* SS18–50 has the great potential to serve as a probiotic for dairy products.

## Graphical abstract

Space mutation is an efficient tool in microbial breeding. We screened out a space flight‐induced mutants of *L. plantarum* with no mutation in 16s RNA sequence, which showed potent probiotic properties and had great potential to serve as a probiotic additive for dairy products.

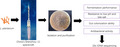

## 1 INTRODUCTION

Probiotics are a group of bacteria that contribute to maintenance or promotion of human health, as they can enhance immune system (Albuquerque‐Souza et al., 2019), alleviate food allergies (Irina, Ceuppens, Seys, Petrova, & Sarah, 2018), and prevent irritable bowel syndrome (Ashton, Malwina, & Davinder, 2018). A desirable probiotic has one or more of the following properties: (a) high resistance to low pH and bile salt during digestion (Jia, 2010), (b) strong adherence to the intestinal cells for colonization (Salminen & Isolauri, 2006), and (c) ability to inhibit pathogens (Coman et al., 2020).

Seeking probiotics with potent probiotic characteristics is of great interest to food and pharmaceutical industries. To reduce chances of failure in isolation and screening of probiotics from natural sources, physical and chemical mutagenesis using ultraviolet or N‐Methyl‐N′‐nitro‐N‐nitrosoguanidine (NTG) is an alternative strategy (Chunli & Zhang, 2010; Yeo & Liong, 2012). With rapid development of space technology, mutagenesis during space flight is one newly developed method for microbial modification. In the harsh outer space with microgravity, various cosmic rays, vacuum and extreme temperature (Hemmersbach, 2015), organisms including microorganisms are prone to genetic mutation (Pei, Hu, Chai, & Zhou, 2019; Wang, 2011). Space exploration can alter many microbial properties including growth rate (Kacena, Manfredi, & Todd), cell envelope (Zea et al., 2017), and secondary metabolites (Gao, Liu, & Zhang, 2011). We recently also selected space flight‐induced *Lactobacillus reuteri,* which showed enhanced anti‐inflammatory property and effectively ameliorated ethanol‐induced gastric injury in rats (M. Sun, Hou, et al., 2018; Sun, Zhang, et al., 2018).

*Lactobacillus plantarum* is a kind of lactic acid bacteria, anaerobic or facultative anaerobic. *L. plantarum* has been reported to have many health beneficial functions *(*Vries, Vaughan, Kleerebezem, & Vos, 2006 *)*. Since space mutagenesis is an efficient strategy for microbial breeding by which we have successfully used in *L. reuteri* screening, in the current study we intended to screen *L. plantarum ‐*a commonly used probiotic in dairy industry, with enhanced probiotic characteristics by space flight mutation.

## 2 MATERIALS AND METHODS

### 2.1 Bacterial strains

*Lactobacillus plantarum GS18* provided by Fullarton Biological Engineering Technology Co., Ltd (Beijing, China) was anaerobically grown in MRS medium at 37°C and used as the wild type sent for space mutagenesis. The pathogens including *Escherichia Coli*, *Staphylococcus Aureus*, *Salmonella Typhimurium,* and *Listeria Monocytogenes* (ATCC) were provided by Jilin Entry‐Exit Inspection and cultured in TSB medium at 37°C with shaking at 100 rpm.

### 2.2 Space mutagenesis

*Lactobacillus plantarum* GS18 was inoculated into semisolid MRS medium containing 0.5% agar in plastic tubes and cultured in a stationary incubator at 37°C for 48 hr under anaerobic condition. One tube was sent to outer space in a special biological bag of the re‐entry capsule by the China's Shenzhou‐11 spacecraft at 07:30am Beijing time on October 17, 2016, and traveled at 393km height for 31 days and 18.5 hr before landing. The other tubes were left on the ground and kept at 4°C as a control in the analysis.

### 2.3 Performance in skim milk fermentation

The bacteria were cultured into MRS broth and streaked on MRS agar plates. Eighty‐eight colonies were randomly selected and cultured for over five generations to assure incapability of reverse mutation before use in this experiment. Each of them (0.5ml) was inoculated in a tube containing 5 ml 12% skim milk and cultured at 37°C. The milk coagulation time and the pH value were recorded. Performance with coagulation time less than 6h and pH less than 4.5 was considered excellent. Sixteen isolates were selected based on their coagulation time and pH of the fermented milk. These isolates were put into skim milk medium and cultured at 37^o^C. After curding, the viscosity was measured by a viscometer (Brookfield LVDV Ⅲ U) under room temperature.

### 2.4 Resistance to low pH and bile salt

The pellet of culture was suspended in either PBS buffer (pH 2.5) and then incubated in an orbital shaker (100 rpm) at 37°C for 3 hr or in PBS buffer (pH 8.0) containing 0.5%(w/v) bile salts (Yuanye Biotechnology, Shanghai, China) which was then incubated in an orbital shaker (100 rpm) at 37°C for 4 hr. The total viable counts were determined by counting colonies on the MRS agar after cultivation at 37°C for 48 hr.

Resistance index (RI) was calculated as follows:

(1) $$\mathrm{RI}={\mathrm{logN}}_{0}/N_{f}$$

N_0_ = initial cell number, N_f_ = final cell number.

### 2.5 Cell surface hydrophobicity

The bacterial solution in PBS buffer was adjusted to 0.8–1.0 (A_0_) at 600 nm_._ The suspension was added with xylene at 3:1 (v/v) and then fully shook for 2 min. After 15 min incubation at ambient temperature, the aqueous phase was carefully sampled and measured at 600 nm using a UV‐2550 spectrophotometer (Shimadzu, Japan). The hydrophobicity (H %) was calculated as follows:

(2) $$H\%=\left(A_{0}‐A\right)/A_{0}\times 100$$

$A_{0}$: initial absorbance; A absorbance after mixing with xylene. The isolates with H % ≥50 were considered to be hydrophobic.

### 2.6 Automatic aggregation

The bacteria in PBS (pH7.2) (OD600 = 0.7–1.0) were cultured in a constant temperature incubator at 37℃. Take 2 ml solution cultured for 6 hr and 12 hr respectively to determine the absorbance value at 600 nm. The automatic aggregation ability was calculated as follows:

(3) $$A\%=(1‐\left(\frac{A_{t}}{A_{0}}\right))\times 100$$

### 2.7 Antibacterial activity

The tested bacteria were cultured in MRS for after 24 hr incubation and then centrifuged at 6,000 g at 4°C for 10 min. The supernatant was adjusted to pH = 6.5 with 1M NaOH and then filtered by a 0.22 μm filter. The filtrate (100 μl) containing tested bacteria was added with 100 μl of 10^5^ CFU/mL specific pathogen (*E. coli, S. aureus, S. typhimurium, and L. monocytogenes*) in a 96‐well microtiter plate and incubated for 24 hr at 37°C in an orbital shaker (100 rpm) before measurement at 600nm. Antibacterial activity was calculated as follows:

(4) $$\%I=\frac{{\mathrm{OD}}_{\text{control}}‐{\mathrm{OD}}_{\text{treatment}}}{{\mathrm{OD}}_{\text{control}}}\times 100$$

${\mathrm{OD}}_{\text{control}}$: absorbance without pathogens; ${\mathrm{OD}}_{\text{treatment}}$: absorbance cocultured with specific pathogen.

### 2.8 16S rDNA gene sequence analysis

The total genomic DNA of the bacteria was extracted using Bacterial Genomic DNA extraction kit (Sangon Biotech, Shanghai, China) and amplified using the universal primers 27F (5’‐AGTTTGATCMTGGCTCAG‐3’) and 1492R (5’‐GGTTACCTTGTTACGACTT‐3’) by polymerase chain reaction (PCR) in a 2,720 Thermal Cycler (Applied Biosystems, Foster City, Calif, USA). The PCR condition was: 94°C for 4 min, followed by 30 cycles of 94°C for 45 s, 55°C for 45 s and 72°C for 1 min and a final extension step of 72°C for 10 min. The PCR product was sequenced by Sangon Biotech (Shanghai, China). The sequences were aligned for comparison between the wild type and mutant.

### 2.9 Statistical analysis

All experiments were performed in replicate or triplicate. The data were presented as mean ± standard deviation (*SD*). The difference was evaluated by Students’ *t* test using IBM SPSS Statistics 19 at significance level at *p*‐value < .05.

## 3 RESULTS AND DISCUSSION

### 3.1 Skim milk fermentation performance of eighty‐eight mutant isolates

Based on milk fermentation assay, 16 isolates of bacteria were selected for further examination, as they showed shorter coagulation time (≤ 6 hr) for milk and lower pH (≤4.5) compared with the wild type (Table 1).

**Table 1:** Aggregation time and pH of milk fermented by different bacteria

Type	Total number	Number of isolates						
		Coagulation time（h)				pH		
		≤6	6–12	13–18	19–24	≤4.5	4.5–5.0	＞5.0
Wild type	1	0	1	0	0	0	1	0
Mutants	88	16	25	21	26	17	61	10

**Figure 1:**
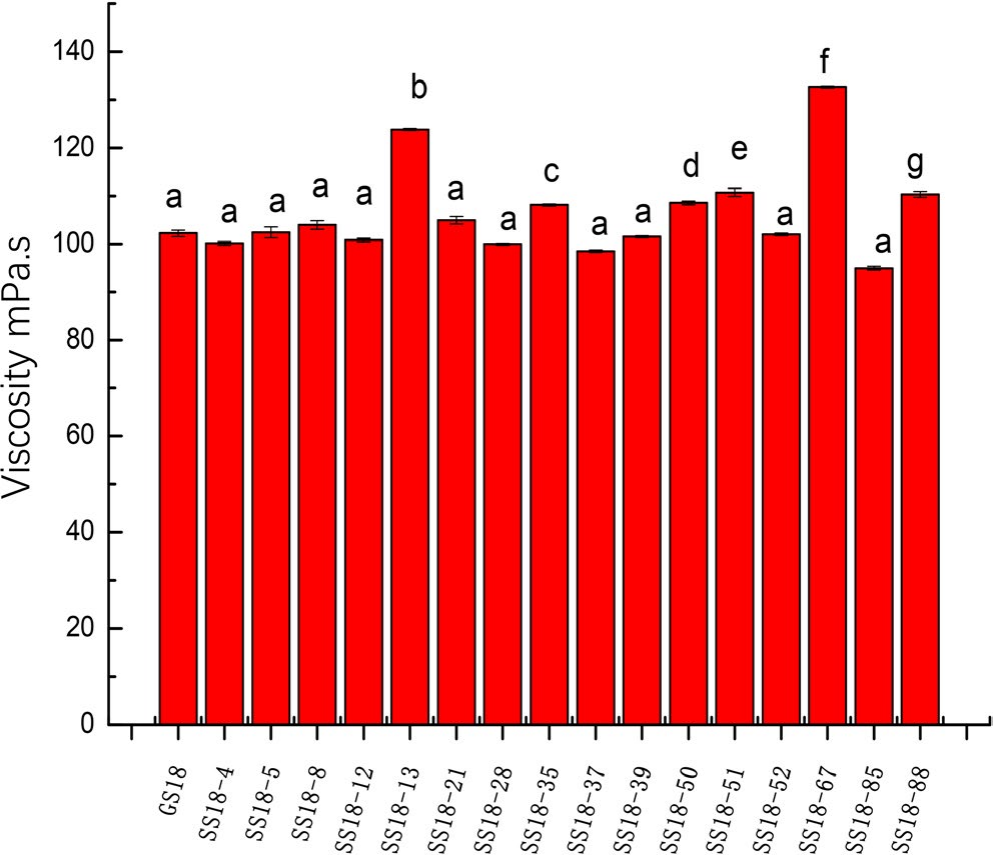
Viscosity of the fermented milk. The viscosity of most bacteria was similar while 6 mutant isolates were significantly higher than that of the wild‐type fermented milk (*p* < .05)

These bacteria were further tested by measuring the viscosity of the fermented milk. The viscosity of skim milk will be increased by the polysaccharides produced in the fermentation process of lactic acid bacteria (Ikeda, Kondoh, Aryantini, Urashima, & Fukuda, 2019). The viscosity of 6 isolates was significantly higher than that of the wild‐type fermented milk (*p* < .05). The six isolates were SS18–13, SS18–50, SS18–51, SS18–67, SS18–35, and SS18–88 (Figure 1).

### 3.2 pH and bile salt tolerance of sixteen mutant isolates showing desirable skim milk fermentation performance

Probiotics have to go through the harsh environment of acid and bile to approach the host gut microbiome (Bezkorovainy & Anatoly, 2001). In general, all selected isolates showed good tolerance to low pH and bile salt compared with the wild type. It is hard to obtain mutants in good tolerance to pH and bile salt by random mutagenesis using traditional methods, whereas space mutagenesis is efficient to generate more beneficial mutations attributable to modification of metabolic capability (Zhang, Fang, & Liu, 2015). After 3 hr of low pH exposure, 11 stains were more resistant to low pH than the wild type. For bile salt resistant, SS18–21 and SS18–50 showed more bile salt tolerant than the wild type, of which only SS18–50 showed both resistance to low pH and bile salt (Table 2).

**Table 2:** Resistance to pH and bile salt

No.	RI（%）	
	pH	Bile salt
GS18	69.80 + 0.49	80.07 + 0.39
SS18–04	86.60 + 0.21*	82.91 + 0.41
SS18–05	－	83.22 + 0.35
SS18–08	－	79.82 + 0.29
SS18–12	76.98 + 0.27*	81.59 + 0.34
SS18–13	76.53 + 0.31*	82.34 + 0.40
SS18–21	－	89.34 + 0.31*
SS18–28	73.22 + 0.44*	84.01 + 0.34
SS18–35	77.40 + 0.52*	80.88 + 0.29
SS18–37	67.66 + 0.31	79.72 + 0.33
SS18–39	79.57 + 0.34*	84.34 + 0.40
SS18–50	75.24 + 0.32*	87.36 + 0.37*
SS18–51	69.66 + 0.31	82.77 + 0.38
SS18–52	77.97 + 0.38*	81.74 + 0.38
SS18–67	76.66 + 0.39*	76.92 + 0.29
SS18–85	78.46 + 0.44*	74.84 + 0.34
SS18–88	75.73 + 0.35*	76.63 + 0.35

* Significantly different compared to wild type at *p* < .05, *n* = 3; －Nondetected.

### 3.3 Hydrophobicity and self‐aggregation activity of sixteen mutant isolates showing desirable skim milk fermentation performance

**Figure 2:**
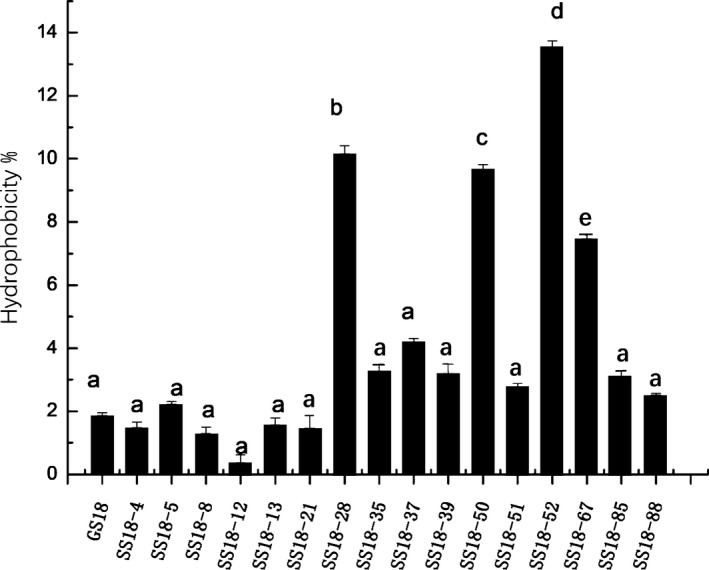
Hydrophobicity of the space mutant isolates. The hydrophobicity of 4 mutant isolates (SS18–28, SS18–50, SS18–51, and SS18–67) had significantly higher hydrophobicity than the wild type GS18 (*p* < .05)

The hydrophobicity and self‐aggregation ability of bacteria are two features that affect the adhesion of bacteria to host tissues (Pascual et al., 2008). The high hydrophobicity of cell surface contributes to the colonization of bacteria on the surface of intestinal mucosa and promotes the adhesion of bacteria to intestinal epithelial cells. The hydrophobicity of 16 mutant isolates to xylene was determined, of which 12 isolates had similar hydrophobicity, and 4 isolates (SS18–28, SS18–50, SS18–51, and SS18–67) had significantly higher hydrophobicity than the standard strain GS18 (*p* < .05). These mutants were considered hydrophobic with H% ranging from 7% to 14% (Figure 2). Adherence to intestinal epithelial cells is an integral probiotic characteristic for transient colonization of probiotics needs the bacteria to adhere to intestinal epithelial cells which is determined by their hydrophobicity (Zhang et al., 2015). The hydrophobicity of *Lactobacillus* strains usually has a good correlation with their adhesion to intestinal epithelial cells (Deepika & Charalampopoulos, 2010; Miljkovic, Thomas, Serror, Rigottier‐Gois, & Kojic, 2019; Vadillo‐Rodríguez, Busscher, Mei, Vries, & Norde, 2005).

**Figure 3:**
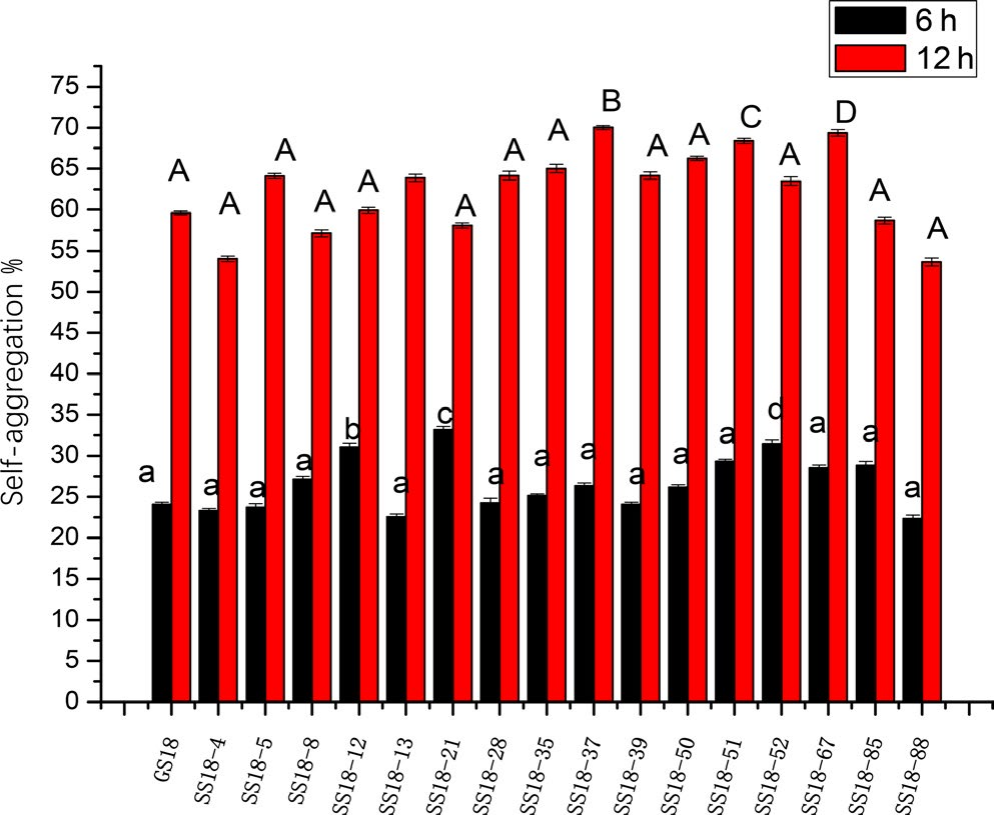
Self‐aggregation ability of the space mutant isolates. All tested bacteria showed good self‐aggregation ability in both 6 hr and 12 hr, of which 3 isolates (SS18–37, SS18–67, and SS18–51) had significantly higher self‐aggregation ability than the standard isolate GS18 (*p* < .05)

The self‐aggregation ability of bacteria is another one of the indicators that determine the adhesion of bacteria to host tissues. All mutant isolates showed good self‐aggregation ability in both 6 hr and 12 hr. Typically, three isolates (SS18–37, SS18–67, and SS18–51) were significantly different from the standard strain GS18 in 12h self‐aggregation (*p* < .05) (Figure 3).

### 3.4 Antibacterial activity of sixteen mutant isolates showing desirable skim milk fermentation performance

Antimicrobial activity against harmful gut pathogens is considered one of the important characteristics of probiotics (Servin & Coconnier, 2003). The supernatants of MRS of most mutant isolates showed powerful antibacterial activity against 4 tested pathogens. Probiotics in the process of culture will produce bacteriocin, which has inhibitory effect on pathogenic bacteria in food. Additionally, lactic acid bacteria like *L. plantarum* can also generate acid microenvironment which can inhibit pathogens (Güven & Benlikaya, 2010). The bacteriostatic effect of 17 isolates was between 20%–30%. Compared with the wild‐type GS18, two isolates (SS18–35 and SS18–50) showed significantly higher antibacterial ability (Table 3). Our research showed that space mutagenesis can improve antimicrobial activity of *L. plantarum*. These findings indicated that the outer space environment may modify some secondary metabolites that inhibit the growth of typical pathogens.

**Table 3:** Antibacterial activity

Isolate	Inhibition rate %			
	*E. coli*	*S. typhimurium*	*S. aureus*	*L. monocytogenes*
*Lactobacillus plantarum* GS18	26.75 ± 0.25	19.68 ± 0.24	22.45 ± 0.23	18.87 ± 0.21
*Lactobacillus plantarum* SS18–04	21.52 ± 0.21	17.97 ± 0.21	24.28 ± 0.34	16.33 ± 0.34
*Lactobacillus plantarum* SS18–05	4.73 ± 0.35	1.08 ± 0.357	0.39 ± 0.27	1.98 ± 0.24
*Lactobacillus plantarum* SS18–08	24.19 ± 0.22	27.21 ± 0.17*	27.04 ± 0.35*	17.29 ± 0.28
*Lactobacillus plantarum* SS18–12	25.23 ± 0.37	19.79 ± 0.44	21.32 ± 0.33	12.82 ± 0.39
*Lactobacillus plantarum* SS18–13	30.51 ± 0.24	21.02 ± 0.36	25.11 ± 0.26	21.54 ± 0.32
*Lactobacillus plantarum* SS18–21	31.66 ± 0.34*	20.59 ± 0.43	20.70 ± 0.44	23.43 ± 0.42*
*Lactobacillus plantarum* SS18–28	32.19 ± 0.26*	21.94 ± 0.29	26.12 ± 0.13	20.41 ± 0.29
*Lactobacillus plantarum* SS18–35	33.02 ± 0.41*	26.30 ± 0.41*	27.01 ± 0.36*	14.89 ± 0.44
*Lactobacillus plantarum* SS18–37	16.50 ± 0.28	14.49 ± 0.21	11.77 ± 0.16	7.312 ± 0.11
*L. plantarum* SS18–39	2.43 ± 0.33	2.10 ± 0.33	10.02 ± 0.22	2.06 ± 0.45
*Lactobacillus plantarum* SS18–50	31.82 ± 0.22*	27.89 ± 0.15*	21.61 ± 0.46	23.9 ± 0.34*
*Lactobacillus plantarum* SS18–51	32.01 ± 0.30*	23.71 ± 0.31	21.13 ± 0.39	22.34 ± 0.45*
*Lactobacillus plantarum* SS18–52	38.44 ± 0.20*	25.88 ± 0.33*	25.14 ± 0.20	15.48 ± 0.35
*Lactobacillus plantarum* SS18–67	24.09 ± 0.39	22.70 ± 0.19	17.40 ± 0.47	9.487 ± 0.44
*Lactobacillus plantarum* SS18–85	30.77 ± 0.18	21.62 ± 0.34	26.88 ± 0.22	11.44 ± 0.21
*Lactobacillus plantarum* SS18–88	4.456 ± 0.25	2.715 ± 0.23	3.83 ± 0.38	1.456 ± 0.36

* Significantly different compared to wild type at *p* < .05, *n* = 3.

### 3.5 16S rDNA sequence comparison of the wild type and the mutant isolate SS18–50

Based on the 16S rDNA sequence, the SS18–50 was identified as the species *L. plantarum*. The 16S rDNA sequence of the wild type and SS18–50 were the same (data not shown). Space mutagenesis did not induce variation of 16S rDNA sequence in our experiment, indicating that 16S rDNA is resistant to space mutagenesis. The enhanced probiotic property was likely attributed to the change of the metabolic pathway.

## 4 CONCLUSION

Space mutagenesis is found to be an effective way for improving probiotic characteristics. The mutant *L. plantarum* SS18–50 was found to possess stronger probiotic characteristics with no variation in 16S rDNA sequence compared to the wild type. *L. plantarum* SS18–50 is a potential probiotic that can be used in fermented food industry.

## CONFLICT OF INTEREST

The authors declare that they have no conflict of interest.

## ETHICAL APPROVAL

This study does not involve any human or animal testing.

5

## Data Availability

The data that support the findings of this study are available on request from the corresponding author. The data are not publicly available due to privacy or ethical restrictions.
